# A Universally Applicable Strategy for Construction of Anti‐Biofouling Adsorbents for Enhanced Uranium Recovery from Seawater

**DOI:** 10.1002/advs.201900002

**Published:** 2019-03-20

**Authors:** Qiuhan Yu, Yihui Yuan, Jun Wen, Xuemei Zhao, Shilei Zhao, Dong Wang, Chaoyang Li, Xiaolin Wang, Ning Wang

**Affiliations:** ^1^ State Key Laboratory of Marine Resource Utilization in South China Sea Hainan University Haikou 570228 P. R. China; ^2^ Institute of Nuclear Physics and Chemistry China Academy of Engineering Physics Mianyang 621900 P. R. China

**Keywords:** anti‐biofouling, marine bacteria, seawater, uranium extraction

## Abstract

The ocean reserves 4.5 billion tons of uranium and amounts to a nearly inexhaustible uranium supply. Biofouling in the ocean is one of the most severe factors that hazard uranium extraction and even cause the failure of uranium extraction. Therefore, development of uranium adsorbents with biofouling resistance is highly urgent. Herein, a strategy for constructing anti‐biofouling adsorbents with enhanced uranium recovery capacity in natural seawater is developed. This strategy can be widely applied to modify currently available carboxyl‐contained adsorbents, including the most popular amidoxime‐based adsorbent and carboxyl metal organic framework adsorbent, using a simple one‐step covalent cross‐link reaction between the antibacterial compound and the adsorbent. The prepared anti‐biofouling adsorbents display broad antibacterial spectrum and show more than 80% inhibition to the growth of marine bacteria. Benefitting from the tight covalent cross‐link, the anti‐biofouling adsorbents show high reusability. The modified amidoxime‐based adsorbents show enhanced uranium recovery capacity both in sterilized and bacteria‐contained simulated seawater. The anti‐biofouling adsorbent Anti‐UiO‐66 constructed in this study exhibits 24.4% increased uranium recovery capacity, with a uranium recovery capacity of 4.62 mg‐U per g‐Ads, after a 30‐day field test in real seawater, suggesting the strategy is a promising approach for constructing adsorbents with enhanced uranium extraction performance.

## 1 Introduction

A sustainable supply of energy remains a cardinal challenge for continuous development by human beings. Currently, with the rapid growth in energy demands and the uncertainty of fossil fuel energy, the use of efficient and clean nuclear energy has attracted increasing attention.^1^ As one of the most critical and effective raw materials for nuclear reactors, uranium (U) plays an indispensable part in the continuous pursuit of alternative energy sources. To make nuclear energy a sustainable energy source, development of economically viable sources of uranium other than land ore is urgently needed in the coming years.^2^ The ocean contains ≈4.5 billion tons of uranium, nearly 1000 times the uranium contents of land ore, making the ocean a potentially large resource for support of nuclear energy production for hundreds of years.^3^ In recent decades, researchers worldwide have tested various strategies for recovering uranium from seawater and aqueous solutions, such as co‐precipitation,^4^ ion exchange,^5^ adsorption,^6^ and organic–inorganic hybrid adsorption.^7^ By using the electroextraction strategy, an extremely high uranium extraction capacity of 1932 mg‐U per g‐Ads was archived by Cui and co‐workers.^8^ Among the diverse methods for uranium recovery, adsorption is a popular method due to its high efficiency, convenient operation, and cost‐effectiveness. Moreover, various materials have been developed as adsorbents for recovery of uranium from seawater, such as polymeric fibers,^9^ biomaterials,^10^ inorganic materials,^11^ porous organic polymers (POPs),^12^ and nanomaterials.^13^

However, biofouling by marine organisms severely limits the performance of the adsorbent used in natural seawater. During practical application of amidoxime‐based fiber adsorbents in natural seawater, biofouling caused a 30% decrease of in the uranium uptake capacity after a 42‐day adsorption test.^14^ Biofouling, which is the accumulation of microorganisms, algae, plants, or animals on a moist surface, severely restricts the application of materials in the ocean environment. In the ocean, biofouling generally passes through four stages, namely, the attachment of an organic membrane, settling of individual bacterial cells and diatoms, formation of a microbial membrane that captures additional particles and large organisms, and the growth of large organisms on the contaminated surface.^15^ The settling of bacterial cell and the formation of a microbial membrane are the most critical steps and are essential for the adhesion of large organisms.^16^ The impact due to biofouling on the properties of uranium adsorbents occurs primarily because the settling of organisms on the adsorbent blocks the ligands used in uranium binding. Additionally, biofouling has a high potential to decrease the reusability of adsorbent by producing protein enzyme that can degrade the adsorbent.^17^ Consequently, to minimize production costs and improve economic efficiency, it is crucially important to develop a uranium adsorbent with biofouling resistance. As a severe threaten to materials used in marine environment, strategy like anticorrosive coating have been used for controlling the marine biofouling.^18^ However, the strategy is not applicable for the adsorbents used for uranium recovery. In recent years, various antifouling coatings have been developed. However, bacterial cells deposited on the surface of the adsorbent cannot be killed. In the construction of antifouling uranium recovery adsorbents, the use of nanoparticles, such as TiO2 nanoparticle and Ag nanoparticles supplied antibacterial activity by killing bacteria.^19^ However, tight cross‐linking is lacking between the antimicrobial substance and the adsorbent, and thus the antimicrobial substance is highly likely to be washed off during the long‐time use in the natural ocean environment. Hence, construction of anti‐biofouling adsorbent by covalent cross‐link seems to be a promising strategy for constructing enhanced adsorbent for uranium recovery from natural seawater.

Due to the large surface area, porous material like metal–organic frameworks (MOFs), COFs, PAFs, POPs are high concerned for uranium extraction.^20^ Among them, the MOF is a crystalline hybrid material that connects metal ions or metal clusters with various organic bridging materials.[qv: 20b,21] Compared with traditional porous materials, such as zeolite molecular sieves and activated carbon, the MOF has high permanent porosity, a tunable pore structure, an extraordinary specific surface area, and adjustable chemical function.^22^ Another unique advantage of MOFs is that they can be used to structurally modify various functional groups or metal ions by in situ synthesis or postmodification, giving MOFs special chemical properties.^23^ Based on these excellent properties, MOFs have become a research hotspot in the field of novelty porous materials and have shown potential applications in adsorption and separation,^24^ catalysis,^25^ molecular sensing and detection,^21^, ^26^ and membrane materials.^27^ Additionally, these materials have recently been demonstrated extensive applications as heavy metal sorbent materials with advantages in sorption kinetics, capacity, and/or selectivity.[qv: 18,20b,21] U(VI) sorption by MOFs was also investigated. For example, Dai and co‐workers reported the first application of UiO‐68 in extraction of actinide elements.^2^ MOF‐76 was used to probe and extract U(VI) from aqueous solution,^28^ and amine‐grafted MIL‐101(Cr) showed enhanced U(VI) sorption capacity.^29^ It is understandable that stability in aqueous solution or acidic media is required for MOFs to serve as sorbents. Lillerud et al. first synthesized a zirconium (IV) dicarboxylate porous material known as UiO‐66,^30^ which is a Zr‐contained metal organic framework material, and this material has attracted extensive attention from researchers worldwide due to its high surface area and unprecedented physicochemical stability. The stability is derived from the highly oxyphilic nature of zirconium (IV) and the SBU (Zr6‐cluster) formed in the MOFs, which makes it highly resistant toward various solvents and high temperature. The aperture is sufficiently large to accommodate uranyl ions. In addition, a series of frameworks with a structure based on the UiO‐66 skeleton were also synthesized, such as UiO‐66‐NH_2_, UiO‐66‐NO_2_, and UiO‐66‐Br.^31^ These previous achievements highlight the vast opportunities for MOFs in uptake and separation of U(VI) from aqueous solutions.

In this study, using the carboxyl group in the adsorbent and a simple one‐step chemical reaction, the broad‐spectrum antibiotic neomycin was covalently cross‐linked with the carboxyl group on the MOF adsorbent UiO‐66, poly(imide dioxime) nanofiber adsorbent PIDO, and amidoxime‐functionalized UHMWPE fiber adsorbent AO, to construct anti‐biofouling uranium adsorption materials. Due to the high antimicrobial activity and the broad spectrum of neomycin, the newly fabricated adsorbents show high inhibition activity against marine bacteria and enhanced uranium uptake capacity in natural seawater. To the best of our knowledge, for the first time, this study constructs anti‐biofouling uranium adsorbents by covalent cross‐linking, which endow the adsorbent with long antimicrobial persistence and high reusability. The strategy is based on the carboxyl group, which widely exists in the amidoxime group‐based adsorbents and selected other carboxyl‐containing adsorbents and can be universally used in modification of the existing amidoxime group adsorbents and other carboxyl group‐containing adsorbents for enhanced uranium recovery performance.

## 2 Results and Discussion

### 2.1 Construction and Characterization of Materials

#### 2.1.1 Construction of Antibacterial UiO‐66

**Figure 1:**
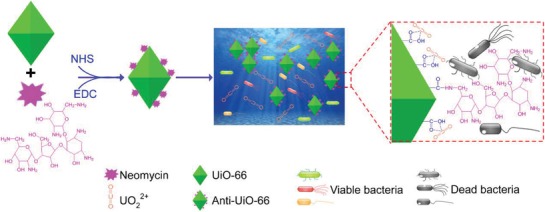
Schematic illustration for construction and function of antibacterial adsorbent Anti‐UiO‐66 for uranium recovery from seawater.

Neomycin is a widely used aminoglycosides antibiotic that inhibits bacteria by inhibiting the protein synthesis or causing collapse of bacterial cell membrane. The antibiotic neomycin shows a broad antibacterial spectrum against Gram positive (G^+^) bacterium, Gram negative (G^−^) bacterium, and mycobacteria.^32^ Because the dominant biofouling bacteria in uranium recovery adsorbents is unclear, a broad‐spectrum antibiotic is a preferable choice for construction of anti‐biofouling adsorbents for uranium recovery. Additionally, the amino group in the aminoglycosides antibiotics is easily covalently cross‐linked with the carboxyl group in the adsorbents. Neomycin contains six amino groups and was chosen for this study (**Figure** **1**). The MOF material UiO‐66 was prepared using terephthalic acid (TPA) and isophthalic acid (IPA), in which IPA is essential for the synthesis of the metal organic framework and IPA is responsible for generation of the carboxyl group for uranium recovery. Based on the reaction of carboxyl group and amino group, the carboxyl group from IPA can react with the amino group from neomycin. To prepare antibacterial UiO‐66, the carboxyl group of the newly synthesized UiO‐66 was cross‐linked with the amino group of neomycin by addition of *N*‐Hydroxysuccinimide (NHS) and (N1‐((ethylimino)methylene)‐N3,N3‐dimethylpropane‐1,3‐diamine) (EDC) to form a peptide linkage. To determine the optimal ratio for cross‐linking of UiO‐66 and neomycin, these two substances were added with the mass ratios (m m^−1^) of 1:0, 1:0.005, 1:0.01, 1:0.02, 1:0.03, 1:0.04, and the antimicrobial activity was determined to choose the optimal antibacterial UiO‐66. The results show that the mixture with a ratio of 1:0.03 generated antibacterial UiO‐66 with the highest antibacterial activity (Figure S1, Supporting Information). This material was selected for following study, and the newly fabricated adsorbent is referred to as Anti‐UiO‐66.

#### 2.1.2 Confirmation of the Successful Construction of Antibacterial UiO‐66

**Figure 2:**
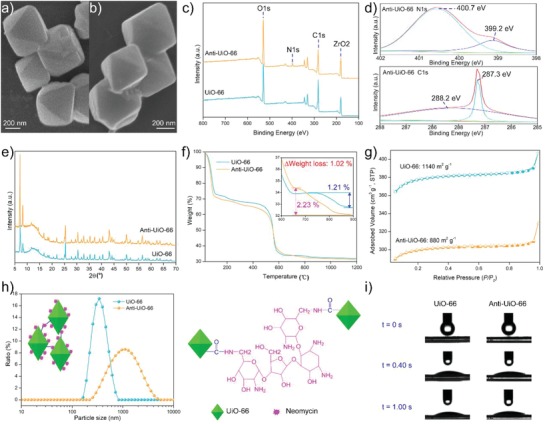
a,b) SEM images, c) XPS spectra, d) high‐resolution spectra of N1s and C1s of Anti‐UiO‐66, e) XRD patterns, f) TGA curves, g) N2 adsorption–desorption isotherms, h) particle size distributions and cross link of nanoparticles, and i) snapshots of the water contact angle test, of UiO‐66 and Anti‐UiO‐66.

The MOF material UiO‐66 is a framework structure, and modification of the material by an additional functional group might cause damage to the crystal structure. Morphology observation of UiO‐66 and Anti‐UiO‐66 using scanning electron microscope (SEM) shows that both the original UiO‐66 and modified UiO‐66 have an octahedron structure, suggesting that the addition of neomycin does not influence the structure of UiO‐66 (**Figure** **2**a,b). The successful coupling of neomycin onto UiO‐66 was determined via X‐ray photoelectron spectroscopy (XPS). The results indicate that the XPS spectra of Anti‐UiO‐66 have one additional peak at 398.4 eV, which corresponds to the core levels of N1s, suggesting the successful cross‐linking of neomycin with UiO‐66 (Figure 2c,d). The N1s spectrum of Anti‐UiO‐66 can be fitted into two peaks centered at binding energies of 399.2 and 400.7 eV, which are attributed to the —NH_2_ and —C—N—H groups, respectively, indicating the successful introduction of the antibiotic neomycin into UiO‐66. The C1s spectrum of Anti‐UiO‐66 can be fitted into peaks of 287.3 and 288.2 eV, corresponding to the —C—O and —N—C=O group, indicating the peptide linkage formed by the reaction of the carboxyl group and amino group. The crystal structure of modified UiO‐66 was further determined using X‐ray diffraction (XRD), and the result revealed that the modified UiO‐66 displayed a structure similar to that of the original UiO‐66 and maintained its crystalline structure (Figure 2e), thus confirmed the findings of the SEM observation.

#### 2.1.3 Characterization of the Antibacterial UiO‐66

The amount of neomycin introduced onto UiO‐66 was determined by thermogravimetric analysis (TGA). The result shows that although UiO‐66 and neomycin are cross‐linked at a mass ratio of 1:0.03, the Anti‐UiO‐66 only contain 1.02% linked neomycin, suggesting that 33.33% of the neomycin does not react with UiO‐66 and that further optimization of the cross‐linking condition can reduce the dosage of neomycin used in construction of the anti‐biofouling uranium adsorbent (Figure 2f). The high surface area of MOF materials is critical to the uranium uptake capacity, whereas the modification of MOF by an additional functional group might cause a decrease in the surface area. The Brunauer–Emmett–Teller (BET) specific surface area indicates that the Anti‐UiO‐66 displayed a lower surface area (880.42 m^2^ g^−1^) than UiO‐66 (1140.72 m^2^ g^−1^), which might occur because the neomycin coupling could block a subset of the pores in UiO‐66 (Figure 2g).The particle size analysis shows that Anti‐UiO‐66 (range from 255 to 4801 nm with an dominant size of 1106 nm) had a larger particle size than the UiO‐66 (range from 164.2 to 712.4 nm with an dominant size of 342 nm), which might occur because the cross‐linking of UiO‐66 particle is mediated by the amino groups from neomycin (Figure 2h). Another key factor affecting the adsorption capacity and kinetics is the hydrophilicity of the adsorbent. As a high hydrophilic material, the introduction of the amino group of neomycin into UiO‐66 might improve the hydrophilicity of the nanoparticles. The hydrophilicity of UiO‐66 and Anti‐UiO‐66 was tested via the water contact angle method. Both UiO‐66 and Anti‐UiO‐66 show high hydrophilicity with contact angel of 18.3° and 17.6° after contact with water drop for 0.4 s, which indicates that the modified of UiO‐66 caused minimal changes in the hydrophilicity (Figure 2i).

### 2.2 Uranium Recovery Capacity

#### 2.2.1 Determination of Optimal pH for Uranium Uptake

**Figure 3:**
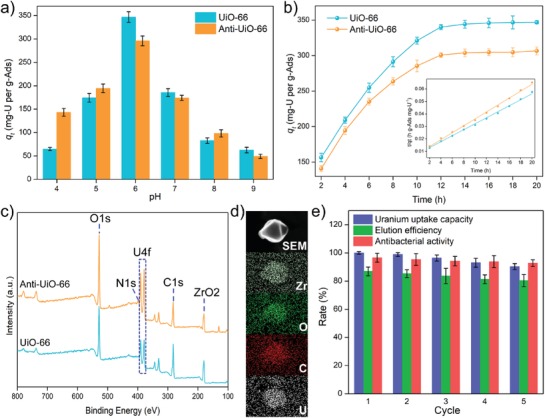
a) Uranium adsorption capacities at different pH, b) uranium adsorption isotherms and pseudo‐second‐order kinetic model, c) XPS spectra, d) SEM images and EDS spectra, and e) reusability, of UiO‐66 and Anti‐UiO‐66.

The pH of the aqueous environment significantly influences the uranium uptake capacity of the adsorbents because the pH of the solution can influence the surface charge of both the adsorbents and the uranyl.^33^ In this study, UiO‐66 and Anti‐UiO‐66 show a similar tendency of benefitting pH for uranium uptake (**Figure** **3**a). In the pH range of 4.0–6.0, the uranium extraction capacity progressively increases but decreases gradually with further increase of the pH to 9.0. The maximum uranium uptake capacities of UiO‐66 and Anti‐UiO‐66 are achieved at pH 6.0 after soaking for 24 h and reach up to 346.89 mg‐U per g‐Ads and 295.95 mg‐U per g‐Ads, respectively. It is notable that Anti‐UiO‐66 shows a decreased uranium uptake capacity than UiO‐66 at all pH environments. The zeta potential of UiO‐66 and Anti‐UiO‐66 in deionized water were also determined and the result shows that these two MOF materials have similar zeta potentials in a neutral environment (Figure S2, Supporting Information), which corresponds with the result showing that these two adsorbents have a similar optimal pH for uranium uptake. Although the loaded neomycin contained six amino group, which might increase the optimal pH of Anti‐UiO‐66, the amount of the loaded neomycin in Anti‐UiO‐66 is low and cause no significantly change to the optimal pH of the adsorbent.

#### 2.2.2 Uranium Recovery Capacity in Uranium Spiked Seawater/Simulate Seawater

Adsorption kinetics analysis shows that UiO‐66 has a higher uranium uptake capacity than Anti‐UiO‐66 (Figure 3b), which might be caused by the following factors: the introduction of antibiotics occupies the functional carboxyl group for uranium adsorption; the surface area of Anti‐UiO‐66 is lower than that of UiO‐66 and maintains a lower surface area for uranium biding; and the pores in UiO‐66 are blocked by the coupling of neomycin, which reduces the entrance of uranium into the pores of Anti‐UiO‐66. The adsorption kinetics of UiO‐66 and Anti‐UiO‐66 are both fit well with the pseudo‐second‐order kinetic mode of adsorption, suggest the adsorption of Anti‐UiO‐66 to uranium is mainly based on chemisorption. The characterizations of uranium loaded adsorbents were also analyzed. A new and strong U4f double peak is observed in the XPS spectra of uranium‐loaded UiO‐66 and Anti‐UiO‐66, which also confirmed the bond of uranium to the adsorbents (Figure 3c). The SEM image shows that the uranium‐loaded Anti‐UiO‐66 maintained an integrated structure, and energy dispersive spectrum (EDS) analysis of uranium‐loaded Anti‐UiO‐66 shows that uranium is bound onto the nanoparticles (Figure 3d). The bonded uranium was also eluted using 0.1 m HNO3 and the result shows that 0.1 m HNO_3_ can washed off 86.31% of the loaded uranium within 150 min, whereas the uranium cannot be eluted by distilled water (Figure S3, Supporting Information). Reusability analysis shows that after adsorption–desorption recycling for 5 cycles, only an average reduction of 2.29% of the uranium uptake capacity and 1.65% of the elution efficiency occur after each cycle (Figure 3e). The reduction of the uranium uptake capacity might be due to damage to the functional group for uranium binding, the blockage of pores in Anti‐UiO‐66 for uranium entry to the inside by tightly bound uranium, and the occupation of functional group by uranium and other tightly bond elements. The reduction of elution efficiency might also be caused by the accumulation of the tightly bond uranium element in Anti‐UiO‐66.

### 2.3 Antibacterial Activity

#### 2.3.1 Antibacterial Spectrum Determination

**Figure 4:**
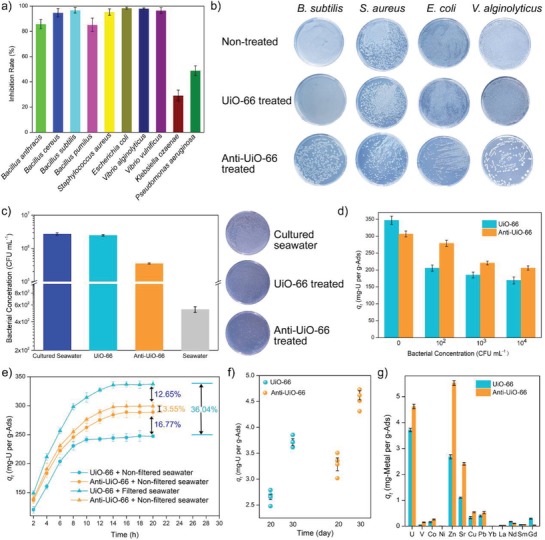
a) Antibacterial spectrum, b) antibacterial activity to indicator bacteria, c) antibacterial activity to marine bacteria, d) uranium uptake capacity in uranium spiked seawater contained different concentration of *V. alginolyticus*, e) uranium uptake capacity in uranium spiked filtered and nonfiltered seawater, f) uranium uptake capacity in natural seawater, and g) specificity to different metal ions in natural seawater, of UiO‐66 and Anti‐UiO‐66.

To evaluate the antibacterial activity and antimicrobial spectrum of Anti‐UiO‐66, the ultraviolet sterilized adsorbent was co‐cultivated with ten bacterial strains from different species at a concentration of 5 mg mL^−1^ in Luria broth (LB) broth and the bacterial concentration were determined after cultivation for 6 h. The inhibition rate was determined using the following equation

(1) $$\mathrm{IR}\;=\;\frac{C_{i}\;-\;C_{a}}{C_{i}}\;\times \;100$$

where *C*_a_ (CFU mL^−1^) indicates the concentration of bacterial cultures treated with adsorbent and *C*_i_ (CFU mL^−1^) indicates the concentration of bacterial cultures without treatment. The result shows that Anti‐UiO‐66 could inhibit the growth of all the ten tested bacterial strain and exhibit inhibition rates greater than 80% for eight of the tested bacterial strains, including 98.01% inhibition rates for the ocean bacterium *Vibrio alginolyticus* (**Figure** **4**a), The addition of UiO‐66 has no significant influence to the growth of the bacteria (Figure 4b). Similar to neomycin, Anti‐UiO‐66 shows a broad antibacterial spectrum and could inhibit the growth of both G^+^ and G^−^ bacteria. The antibacterial activity of Anti‐UiO‐66 to the marine bacteria was also determined, and the result reveals that Anti‐UiO‐66 showed 87.03% inhibition to the growth of marine bacteria, but no significant inhibition activity is observed for UiO‐66 (Figure 4c). The same as the antibacterial mechanism of neomycin, Anti‐UiO‐66 might exert antibacterial activity by causing the collapse of the bacterial cell leading to the death of bacterial cell.^34^

#### 2.3.2 Uranium Adsorption in Bacteria‐Containing Environments

Marine microorganisms are an important component of the marine ecosystem. Thus, biofouling of the adsorbent is inevitable during the process of uranium extraction from natural seawater. To explore the influence of bacteria on the adsorption capacity of the adsorbent to uranium, the marine bacterium *V. alginolyticus* was chosen as simulative environmental bacteria. Adsorption experiments were conducted in uranium‐spiked stimulate seawater with a uranium concentration of 8 ppm and exponential‐growth‐phase *V. alginolyticus* was added to the solution for final concentrations of 10^2^, 10^3^, 10^4^ CFU mL^−1^, respectively. The concentration of bacteria has a significant influence on the uranium extraction properties of adsorbents. Compare with UiO‐66 soaked in uranium‐spiked seawater without addition of bacteria, the uranium uptake capacity of UiO‐66 soaked in uranium spiked seawater containing 10^2^ CFU mL^−1^ of strain *V. alginolyticus* reduces by 40.75% and reduces progressively accompany with the increase of bacterial concentration (Figure 4d). However, the adsorption performance of Anti‐UiO‐66 is less affected by the addition of bacteria than UiO‐66 and a reduction of only 9.15% in uranium uptake capacity is observed for Anti‐UiO‐66 soaked in uranium‐spiked seawater containing 10^2^ CFU mL^−1^ of strain *V. alginolyticus*. The uranium uptake of Anti‐UiO‐66 is 35.74% higher than that of UiO‐66 soaked in uranium‐spiked seawater containing 10^2^ CFU mL^−1^ bacterial cells. With the increase in bacterial concentration, which actually occurs in long‐term field test, the uranium uptake capacity is further reduced.

Moreover, to further test the uranium adsorption performance in real uranium‐ spiked seawater, which contains ≈539 CFU mL^−1^ marine bacterium (Figure 4c), the adsorption experiments were conducted with filtered and nonfiltered seawater to simulate bacteriological and aseptic environments. The uranium was added to the filtered and unfiltered seawater to a final concentration of 8 ppm. The result shows that the filtering of seawater causes an increase of uranium uptake capacity for UiO‐66 of 36.04%, and only a slightly increase of 3.55% is observed for Anti‐UiO‐66 (Figure 4e). In the filtered uranium‐spiked seawater, the uranium uptake capacity of Anti‐UiO‐66 is 12.65% lower than that of UiO‐66. However, in the nonfiltered uranium‐spiked seawater, the uranium uptake capacity of Anti‐UiO‐66 reached up to 284.45 ± 4.56 mg‐U per g‐Ads and was 16.77% higher than the uranium uptake capacity of UiO‐66, which was 244.25 ± 5.56 mg‐U per g‐Ads.

Due to the lack of tight covalent cross‐link, previously developed anti‐biofouling uranium adsorbent showed lower reusability and the antibacterial activity was sharply reduced after reuse. However, after reuse for 5 cycles, only an average reduction of 0.9575% of the antibacterial activity is observed (Figure 3e), suggesting that the tight covalent cross‐linking is much more stable for construction of anti‐biofouling adsorbents.

#### 2.3.3 Uranium Uptake Capacity in Natural Seawater

The uranium uptake capacities of Anti‐UiO‐66 and UiO‐66 were also determined in natural seawater without additional uranium. In brief, 5 mg of adsorbents were soaked in 10 L natural seawater with moderated stirring. After soaking for 30 days, the soaked adsorbents were collected, and the amount of loaded uranium were determined by inductively coupled plasma mass spectrometry (ICP‐MS). The result shows that UiO‐66 exhibits a uranium uptake capacity of 3.71 ± 0.07 mg‐U per g‐Ads, and the uranium uptake capacity of Anti‐UiO‐66 is 4.62 ± 0.09 mg‐U per g‐Ads, which is 24.4% higher than that of UiO‐66 (Figure 4f), suggesting that the introduction of antibacterial activity can also significantly increase the uranium uptake capacity of adsorbent in natural seawater. The specificities of the adsorbents to different metal ions in natural seawater have also been determined. The result shows that both UiO‐66 and Anti‐UiO‐66 show high uptake capacity to Zn, U, and Sr (Figure 4g), which was corresponding to previously study on the specificity of UiO‐66 to different metal ions.^35^ The introduction of antibacterial compounds also increases the uptake capacity to the other metals.

### 2.4 Universal Applicable of the Strategy

**Figure 5:**
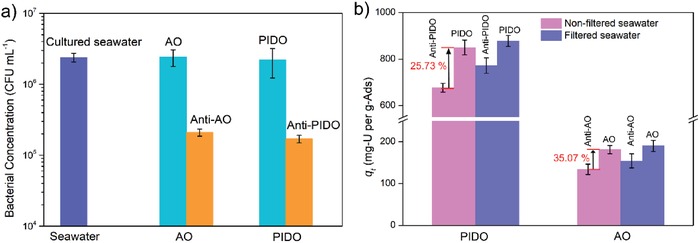
a) Antibacterial activity and b) uranium adsorption capacity of anti‐biofouling PIDO and AO fibers.

The strategy used in this study is based on the covalent cross‐linking between carboxyl group from the adsorbents and amino group from the antimicrobial compounds. According to previous reports, the amidoxime group‐based uranium adsorbent, which is most popular and reliable adsorbent, contains carboxyl group after the amidoximate process.^36^ Furthermore, the treatment of amidoxime group‐based adsorbent with an alkaline substance, which is essential for enhancing the performance of uranium uptake capacity, could also generate additional carboxyl group by the hydrolyze of nitrile group.^37^ Therefore, the strategy developed in this study can be applied for modification of the amidoxime group‐based adsorbents for uranium recovery. In this study, neomycin was introduced into two different types of fiber adsorbents, namely, and PIDO nanofiber^38^ and amidoximate functionalized UHMWPE fiber (AO) fiber,^39^ to demonstrate the universal applicable of the strategy. The introduction of neomycin to PIOD and AO causes no significant changes to the morphologies of these two types of adsorbents (Figure S4, Supporting Information). The results show that after a simple one‐step reaction, both the PIDO nanofiber and AO fiber display inhibitive activity to the marine bacteria and shows 80.9% and 86.6% inhibition to the growth of marine bacteria, respectively (**Figure** **5**a). The uranium uptake capacities in nonfiltered uranium‐spiked seawater are higher than that of the fiber without loaded neomycin by 20.39% and 25.93%, for the PIDO nanofiber and AO fiber, respectively (Figure 5b). In filtered seawater, after the introduction of neomycin, the uranium uptake capacity of PIOD and AO fiber also increased by 13.81% and 23.33%, respectively. One interesting finding was that after the introduction of neomycin to the amidoxime‐based fiber adsorbents, the uranium uptake capacities of the fibers are increased both in filtered seawater and nonfiltered seawater. The neomycin contained amino group and hydroxy group, which might contact with the uranyl ions in the uranium spiked seawater for uranium uptake and increase the hydrophilicity of the fiber adsorbent. For the MOF‐based adsorbents, the carboxyl is critical for uranium adsorption. However, for the amidoxime‐based adsorbents, the amidoxime is critical for the uranium coordination with uranyl, while the carboxyl is only functional for increasing the hydrophilicity. Thus, the introduction of neomycin to the amidoxime‐based adsorbent not only causes any influence to the functional group for uranium uptake, but also provides more functional group for uranium uptake and increases the hydrophilicity to the adsorbent, which endows the modified adsorbents with higher uranium uptake capacity in both bacterial‐contained condition and aseptic condition. After loading of uranium, the fiber color of Anti‐AO and Anti‐PIDO changed from pale yellow to dark orange (Figures S5 and S6, Supporting Information) and the loaded uranium could be observed by EDS analysis (Figure S7, Supporting Information).

Due to the different features of the materials used in ocean, the dominant biofouling bacteria are different.[qv: 19]a,c Though the neomycin used in this study shows a broad antimicrobial spectrum, the constructed anti‐biofouling adsorbent cannot inhibit the growth of all marine bacteria because certain of the marine strains are resistant to neomycin. Thus, the individual antimicrobial compounds that target the specific biofouling bacteria of the adsorbents might be much more efficient for construction of anti‐biofouling adsorbents. The amino group also widely exists in the antimicrobial compounds, including antibiotics, antimicrobial peptides, and proteins with antimicrobial activity, and these antimicrobial compounds show a diverse antimicrobial spectrum.^40^ Thus, by changing the antimicrobial compound that contain amino group, the strategy developed in this study is universally applicable for constructing anti‐biofouling uranium adsorbents of different natures with individual targeted bacteria.

## 3 Conclusion

This study developed a universal applicable strategy for constructing anti‐biofouling adsorbents with enhanced uranium recovery in natural seawater. The strategy is based on a one‐step simple reaction between the carboxyl group in the adsorbents and the amino group in the antimicrobial compounds. Based on further individual demands for anti‐biofouling activity to specific bacterium, the adsorbent could be modified with this strategy by changing the antimicrobial compound that contain amino group. Using the carboxyl‐containing MOF material UiO‐66, an anti‐fouling uranium extraction adsorbent with enhanced uranium uptake capacity in bacteria‐contained seawater was developed. The anti‐fouling adsorbent Anti‐UiO‐66 shows 24.4% increased uranium uptake capacity compared with UiO‐66 after a field test in natural seawater for 30 days. Due to the covalent cross‐linking of the antimicrobial compound with the adsorbent, the antibacterial activity can be retained after long‐time reuse in seawater, which could reduce the economic cost of uranium extraction. The strategy was also used in modification of amidoxime group‐based fiber adsorbents PIDO and AO, and the modified anti‐biofouling fiber adsorbents showed increased uranium uptake capacity in bacteria‐containing uranium‐spiked seawater and high antibacterial activity to marine bacteria. In conclusion, the universal applicable strategy for constructing anti‐biofouling adsorbent developed in this study is a promising approach for enhancing uranium recovery performance and reducing the economic cost for uranium extraction from seawater.

## 4 Experimental Section

*Preparation of Materials*: To prepare MOF nanoparticles of UiO‐66, ZrCl_4_ (1.165 g), TPA (0.665 g), IPA (0.166 g), and CH_3_COOH (9.006 g), were completely dissolved in DMF (37.64 g) by ultrasonic (at a molar ratio of 1 ZrCl_4_/0.8 TPA/0.2 IPA/30 CH_3_COOH/103 DMF) according to previous reports.^41^ The mixed precursor was transferred to a 100 mL Teflonlined autoclave and reacted for 24 h at 120 °C. After cooling, the synthesized solid product was obtained by centrifugation (8000 rpm, 15 min), washed three times with DMF and methanol, and finally dried under vacuum for 12 h at 60 °C. The product is referred to as UiO‐66. Subsequently, UiO‐66 (0.12 g) and EDC (0.06 g) were dispersed in an MES (10 mL) solution of 4 mg mL^−1^. The MES solutions (10 mL) containing neomycin (0.0036 g) and NHS (0.04 g) were added to above reaction system. The resulting mixture was oscillated at room temperature for 24 h in a constant temperature rocker at 25 °C. The treated UiO‐66 was removed from the solution via centrifugation (8000 rpm, 15 min) and rinsed exhaustively with ultrapure water more than five times (with a duration of 10 min each time) to remove unreacted free radicals. The solution was dried in a vacuum oven at 50 °C. This product is referred to as Anti‐UiO‐66. The PIDO nanofiber^38^ and AO fiber^39^ were prepared as previously described, and the neomycin was cross‐linked with the PIDO nanofiber and AO fiber using the same method as described above.

*Characterization of Materials*: The microstructures of the UiO‐66/Anti‐UiO‐66 were observed using a Hitachi S‐4800 scanning electron microscope, and EDS analysis was performed using a Bruker Nano XFlash Detector 5030. XRD analysis of the materials was conducted using a Bruker AXS Diffractometer D8 instrument. The FTIR spectra of the materials were analyzed with a PerkinElmer FTIR spectrometer. The N2 adsorption–desorption isotherms were measured using a Micromeritics ASAP 2460 physical adsorption instrument, the BET specific surface area was accordingly calculated at the relative pressure (P/P0) from 0.01 to 1.0, and the total pore size and pore volume were determined at a P/P0 of 0.99. To test the water contact angle of the prepared materials, the materials were tableted, and the contact angles were determined by using a contact angle meter. A Kratos AXIS‐SUPRA spectrometer was used in XPS of the materials. The zeta potentials of the materials were analyzed using a Zetasizer Nano S90.

*Uranium Adsorption in Uranium Spiked Seawater*: The uranium uptake capacity assay was performed by adding of 10 mg adsorbent into 1 L 8 ppm uranium spiked stimulate seawater with a pH of 6.0 by using a magnetic stirrer with a speed of 100 rpm at room temperature. The stimulated seawater was comprised of 438.607 × 10^−3^ m sodium chloride and 2.297 × 10^−3^ m sodium bicarbonate in ultrapure water. To determine the optimal pH for uranium uptake, a batch of 48 mL uranyl nitrate stock solutions (1000 ppm of U) were diluted with stimulate seawater to obtain 6 L of 8 ppm uranium solution for six samples. The pH of the solution was adjusted using sodium hydroxide and hydrochloric acid solution to 4.0, 5.0, 6.0, 7.0, 8.0, and 9.0, respectively. The adsorbent (10 mg) was added into a 1 L bottle with 8 ppm uranium‐spiked stimulate seawater and shaken for 24 h until the adsorption equilibrium was reached. An aliquot was removed and analyzed using inductively coupled plasma optical emission spectrometry (ICP‐OES). The amount of uranium uptake by adsorbent was calculated using the following formula

(2) $$q_{t}\;=\;\frac{\left(C_{0}\;-\;C_{t}\right)\;\times \;V}{m}$$

where *t* is the contact time, *q_t_* (mg‐U per g‐Ads) represents the adsorption amount of uranium after a contact time of *t*, *C*_0_ (mg L^−1^) is the initial uranium concentration, *C_t_* (mg L^−1^) is the uranium concentration at time *t*, *V* (L) is the volume of the used uranium solution, and *m* (g) is the mass of the adsorbent used.

*Antibacterial Activity Assay*: The dilution plate counting method was used according to the Chinese standard GB/T20944 to determine the antibacterial properties of the adsorbent.[qv: 19c] In brief, sterilized LB solid medium was poured into the aseptic plates to prepare sterile plate count agar plates under aseptic conditions. The bacterial strains *Bacillus anthracis* strain A16R, *Bacillus cereus* strain ATCC 10987, *Bacillus subtilis* strain 168, *Bacillus pumilus* strain GR8, *Staphylococcus aureus* strain TAO‐1, *Escherichia coli* strain BL‐21, *V. alginolyticus* strain CICC 10889, *V. vulnificus* strain CICC 21615, *Klebsiella ozaenae* strain 02116, and *Pseudomonas aeruginosa* strain PA01 were used to test the antimicrobial spectrum of the adsorbents. The exponential growth bacteria were transferred into fresh LB broth with a volume of 5 mL at a ratio of 1% (V V^−1^), and the adsorbents were added into the medium at a ratio of 0.05% (m V^−1^). After cultivated for 6 h at 37 °C with moderate shaking (180 rpm), the bacterial cultures were collected to determine the concentration of the bacteria using the dilution plate counting method. The inhibition rate was calculated using Equation (1).

*Sorption and Antibacterial Assay in Uranium‐Spiked Seawater*: Seawater collected from the South China sea near the Boundary Island was divided into two groups (filtered group and unfiltered group) to mimic bacteria and sterile environment. Sterile seawater was obtained by filtering through a 0.22 µm filter to remove insoluble particles and microorganisms. To further investigate the effect of bacteria on the adsorption of uranium, the adsorption properties of UiO‐66 and Anti‐UiO‐66 were analyzed in the bacterial environment and the sterilized environment. The strain *V. alginolyticus* was added to the sterile seawater to final concentration of 10^2^, 10^3^, and 10^4^ CFU mL^−1^ and the uranyl nitrate was added to a final concentration of 8 ppm. The pH of the solution was adjusted to 6.0 after the addition of uranyl nitrate. For each sample, 10 mg adsorbent and 1 L uranium‐spiked seawater were used in the test. At an interval of 2 h, an aliquot was removed and the uranium concentration was analyzed by ICP‐OES. The amount of uranium uptake by UiO‐66 and Anti‐UiO‐66 was calculated using Equation (2).

*Reusability Assay*: To elute the loaded uranium on the adsorbent, deionized water, 0.1 mol L^−1^ nitric acid solution, and oscillation were used.^42^ The concentration of eluted uranium in the elution solution was determined every 30 min via ICP‐OES. The elution efficiency was calculated using the following formula

(3) $$\mathrm{EE}\;=\;\frac{C_{\mathrm{et}}\;\times \;V_{e}}{q_{e}\;\times \;m}$$

where *C*_et_ (mg L^−1^) indicates the concentration of uranium in the elution solution at elution time *t*, *V*_e_ (L) indicates the volume of the elution solution, *q*_e_ (mg‐U per g‐Ads) indicates the equilibration amount of uranium after the adsorption process, and *m* indicates the mass of used adsorbent. To determine the reusability of the anti‐biofouling adsorbents, 10 mg of adsorbent was soaked in 1 L 8 ppm uranium‐spiked seawater with a pH of 6.0 for 24 h, and the uranium uptake capacity was determined using Equation (2). Subsequently, the uranium‐loaded Anti‐UiO‐66 was eluted with 30 mL 0.1 mol L^−1^ nitric acid solution at room temperature, and centrifuged for 8 min to remove the adsorbents. Finally, the concentration of the eluted uranium in the supernatant was detected by ICP‐OES and the elution efficiency was determined using Equation (3).

*Uranium Extraction in Natural Seawater*: The uranium uptake capacity of Anti‐UiO‐66 and UiO‐66 were also determined in natural seawater without the addition of additional uranium. Generally, amounts of 5 mg of adsorbents were soaked in 10 L natural seawater with moderated stirring. After soaking for 20 and 30 days, respectively, the soaked adsorbents were collected, and the amounts of loaded uranium were determined by ICP‐MS.

## Conflict of Interest

The authors declare no conflict of interest.

## Supporting information

Supplementary
