# Supplementary material for: A Universally Applicable Strategy for Construction of Anti‐Biofouling Adsorbents for Enhanced Uranium Recovery from Seawater
Source: Adv Sci (Weinh). 2019 Mar 20;6(13):1900002. doi: 10.1002/advs.201900002 (PMC6662298; doi:10.1002/advs.201900002)
Supplement: Supplementary file 1 — Supplementary [file ADVS-6-1900002-s001.pdf]

Copyright WILEY-VCH Verlag GmbH & Co. KGaA, 69469 Weinheim, Germany, 2019.

ADVANCED SCIENCE
Open Access

Supporting Information

for *Adv. Sci.,* DOI: 10.1002/advs.201900002

A Universally Applicable Strategy for Construction of Anti-Biofouling Adsorbents for Enhanced Uranium Recovery from Seawater

Qiuhan Yu, Yihui Yuan, Jun Wen, Xuemei Zhao, Shilei Zhao, Dong Wang, Chaoyang Li, Xiaolin Wang, and Ning Wang**

Copyright WILEY-VCH Verlag GmbH & Co. KGaA, 69469 Weinheim, Germany, 2019.

Supporting Information

Universally Applicable Strategy for Construction of Anti-biofouling Adsorbents for Enhanced Uranium Recovery from Seawater

Qiuhan Yu, Yihui Yuan*, Jun Wen, Xuemei Zhao, Shilei Zhao, Dong Wang, Chaoyang Li, Xiaolin Wang, Ning Wang*

Q. Yu, Dr. Y. Yuan, X. Zhao, S. Zhao, Dr. D. Wang, Prof. C. Li, Prof. N. Wang

State Key Laboratory of Marine Resource Utilization in South China Sea

Hainan University

Haikou 570228, P. R. China

Email: yuanyh@hainu.edu.cn; wangn02@foxmail.com

Dr. J. Wen, Prof. X. Wang

Institute of Nuclear Physics and Chemistry

China Academy of Engineering Physics

Mianyang 621900, P. R. China

Figures and Tables

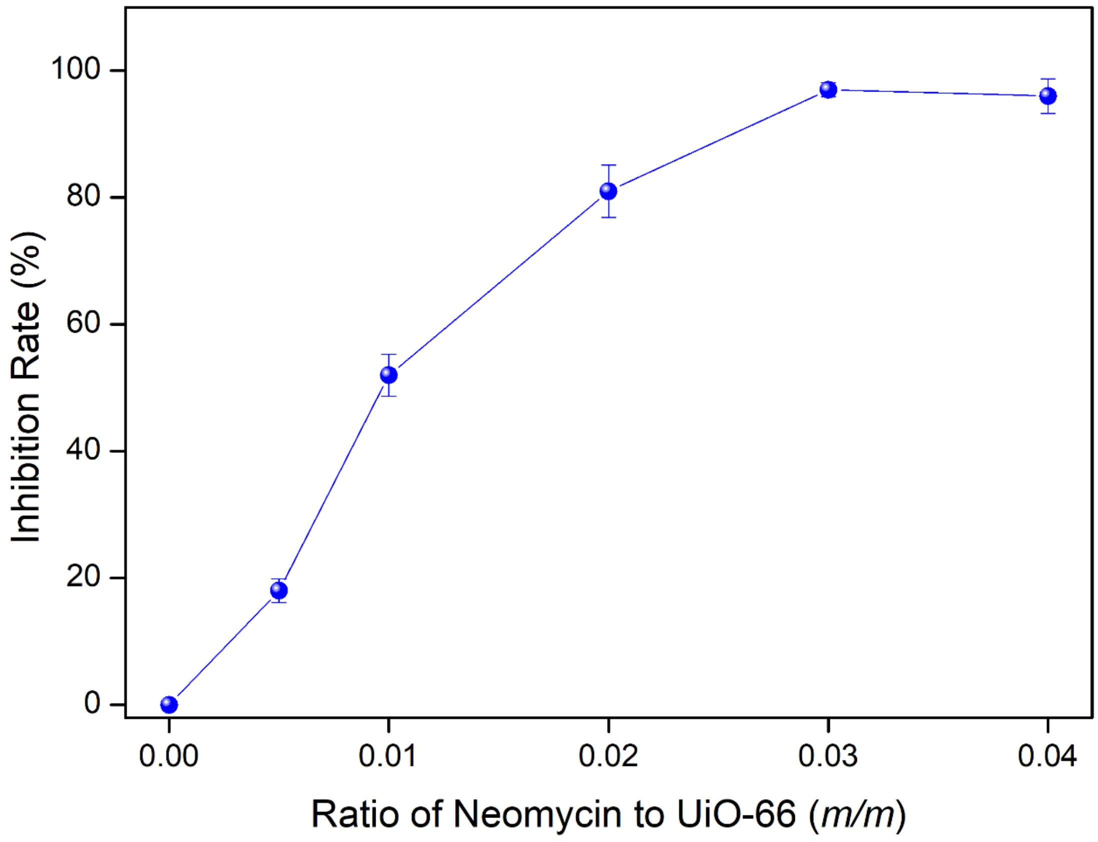

Figure S1. Antibacterial activity of UiO-66 fabricated with different ratios of neomycin.

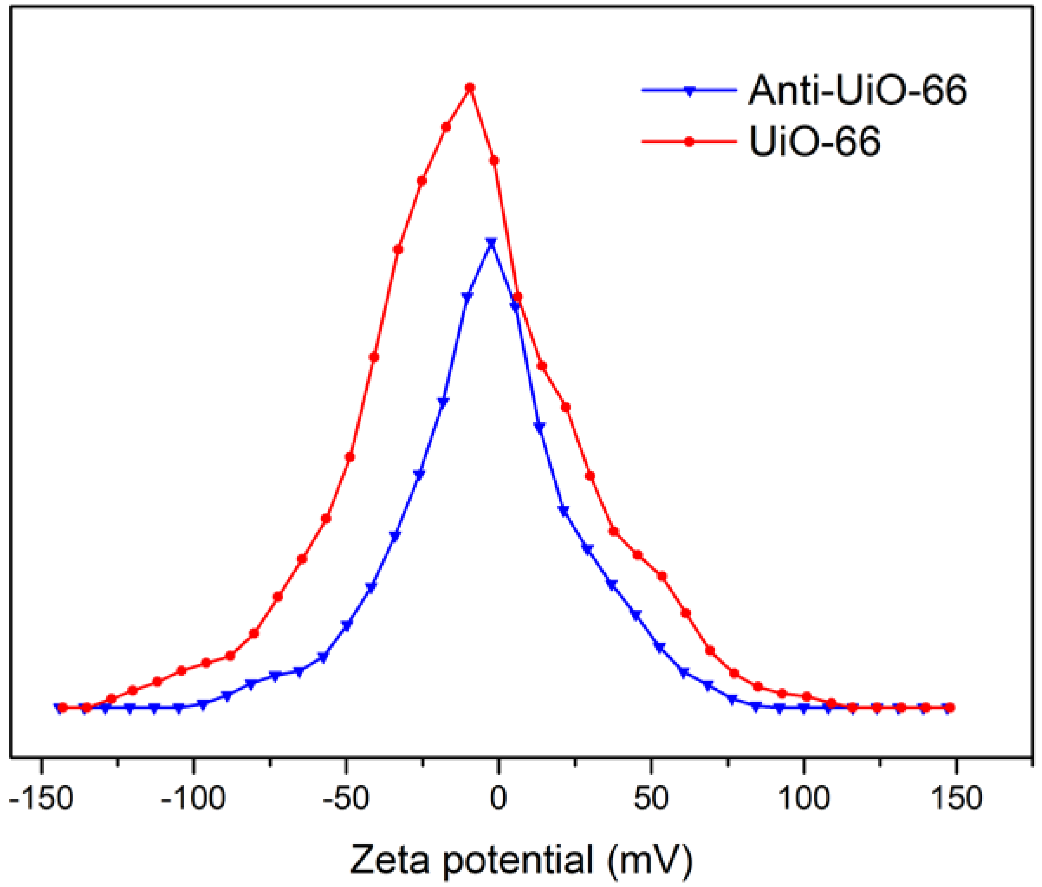

Figure S2. Zeta potential of UiO-66 and Anti-UiO-66 in deionized water

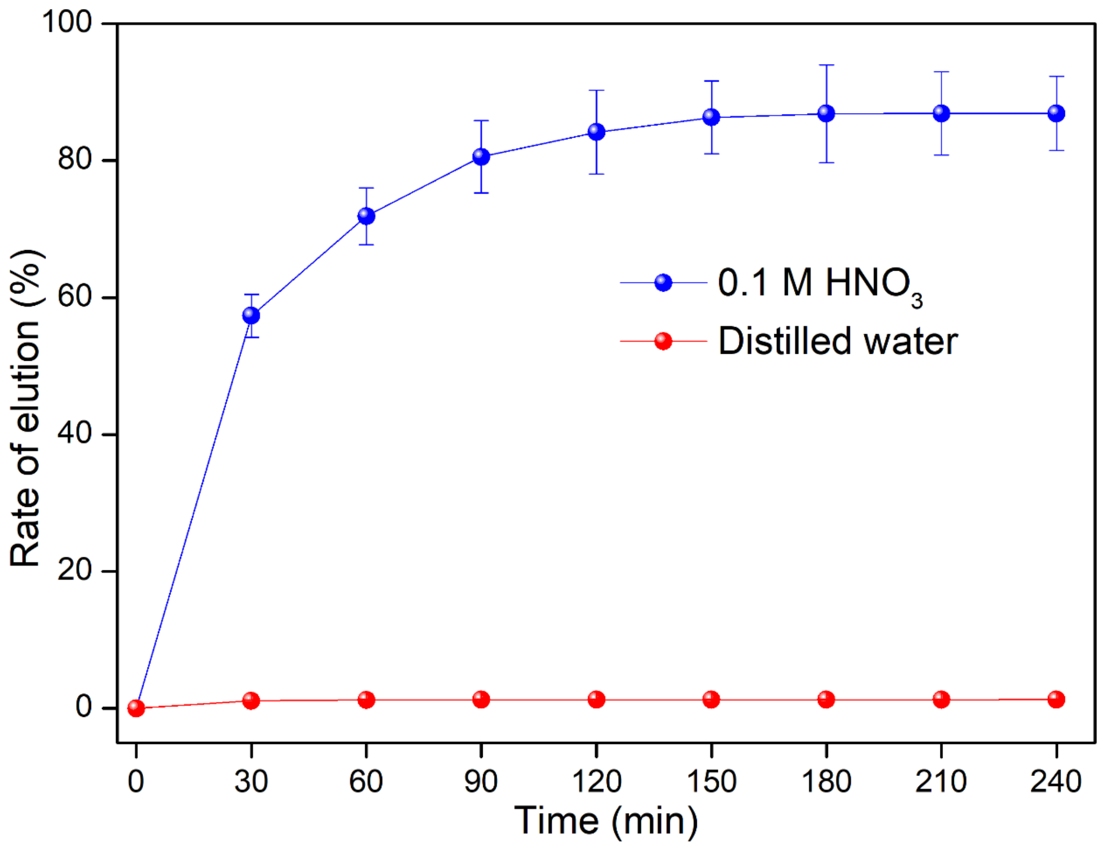

Figure S3. Elution uranium form uranium-loaded Anti-UiO-66.

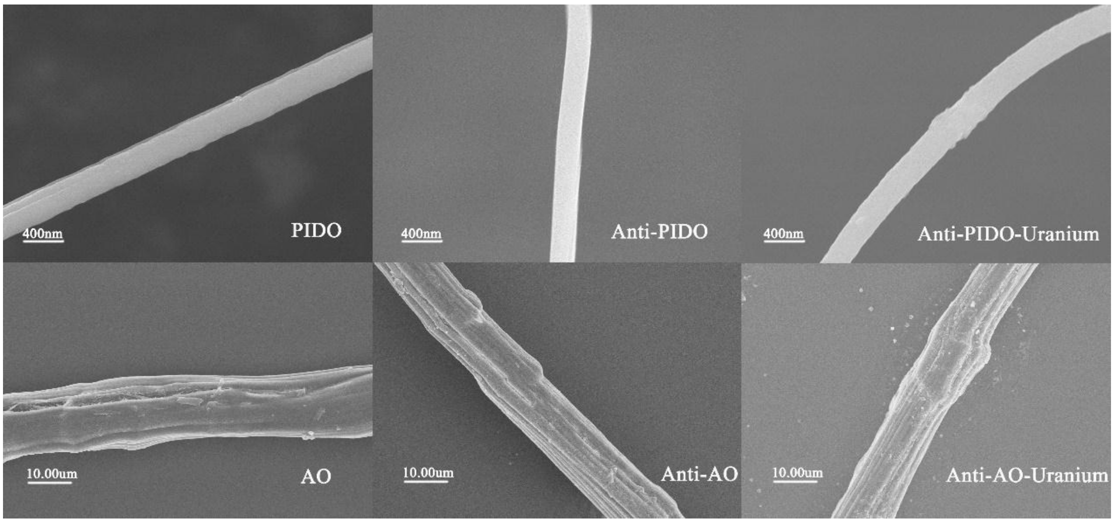

Figure S4. SEM of PIDO (PIDO, Anti-PIDO, Anti-PIDO-Uranium) and AO (AO, Anti-AO, Anti-AO-Uranium)

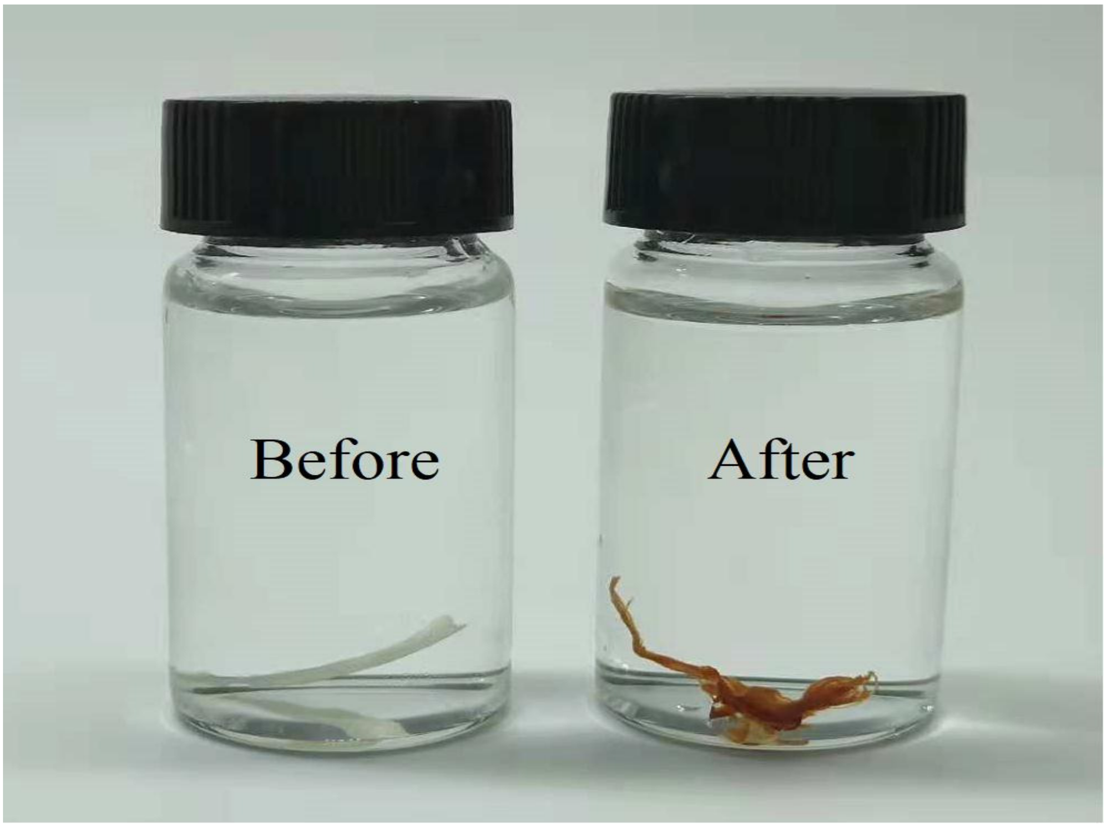

Figure S5. Morphology of Anti-PIDO before and after uranium loading.

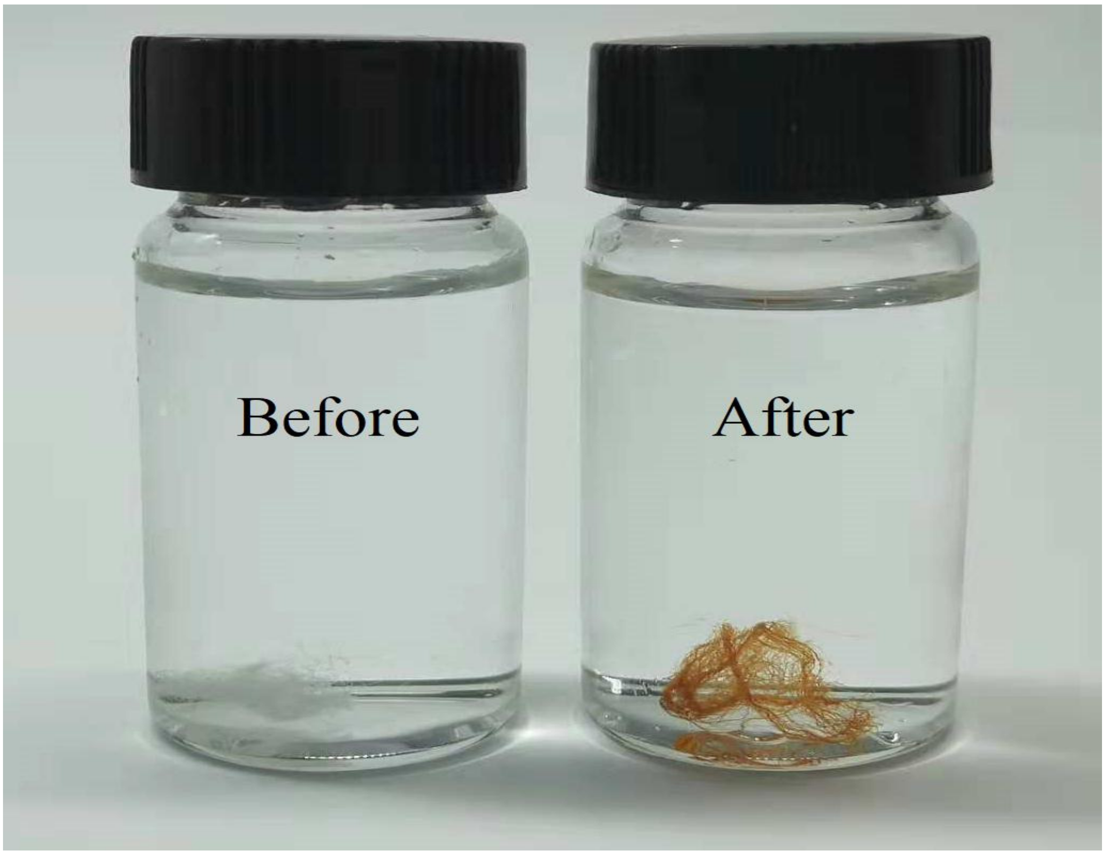

Figure S6. Morphology of Anti-AO before and after uranium adsorption.

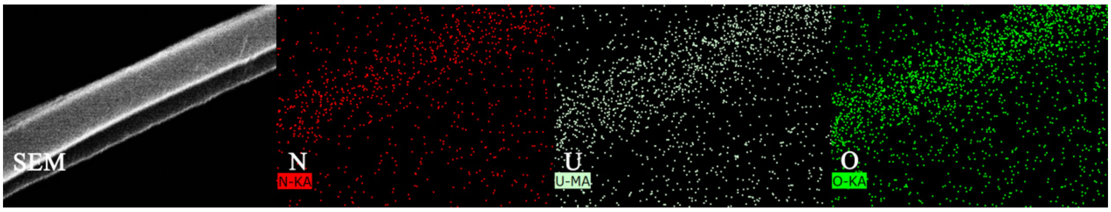

Figure S7. SEM image and EDS analysis of Anti-PIDO after adsorption
